# Geometry and Entanglement of Two-Qubit States in the Quantum Probabilistic Representation

**DOI:** 10.3390/e20090630

**Published:** 2018-08-24

**Authors:** Julio Alberto López-Saldívar, Octavio Castaños, Eduardo Nahmad-Achar, Ramón López-Peña, Margarita A. Man’ko, Vladimir I. Man’ko

**Affiliations:** 1 Instituto de Ciencias Nucleares, Universidad Nacional Autónoma de México, Apdo. Postal 70-543, Mexico City 04510, Mexico; 2 Moscow Institute of Physics and Technology (State University), Institutskii per. 9, Dolgoprudnyi, Moscow Region 141700, Russia; 3 Lebedev Physical Institute, Russian Academy of Sciences, Leninskii Prospect 53, Moscow 119991, Russia; 4 Department of Physics, Tomsk State University, Lenin Avenue 36, Tomsk 634050, Russia

**Keywords:** quantum entanglement, geometric representation of qudits, probability distributions, linear entropy, Bell states

## Abstract

A new geometric representation of qubit and qutrit states based on probability simplexes is used to describe the separability and entanglement properties of density matrices of two qubits. The Peres–Horodecki positive partial transpose (ppt) -criterion and the concurrence inequalities are formulated as the conditions that the introduced probability distributions must satisfy to present entanglement. A four-level system, where one or two states are inaccessible, is considered as an example of applying the elaborated probability approach in an explicit form. The areas of three Triadas of Malevich’s squares for entangled states of two qubits are defined through the qutrit state, and the critical values of the sum of their areas are calculated. We always find an interval for the sum of the square areas, which provides the possibility for an experimental checkup of the entanglement of the system in terms of the probabilities.

## 1. Introduction

The states of quantum systems are determined by wave functions [1,2] (pure states) or density matrices [3,4]. The corresponding definition of these states is done by using state vectors or density operators in the Hilbert space [5]. For qudits, we discuss the approach where the quantum states are identified with fair probability distributions. Different quasiprobability representations of the density operators, such as the Wigner function [6], Husimi *Q*-function [7] or the Glauber–Sudarshan *P*-function [8,9], were introduced to describe continuous variable quantum systems. These functions have been also defined for discrete variable systems such as spin-1/2 particles [10]. In addition, the formulation of quantum states without probability amplitudes was proposed in [11], and the geometric definition of the quantum state determined by the transition probabilities was presented in [12].

Recently, the probability representation of quantum states was introduced both for continuous variables [13] and spin systems [14,15]. This approach uses quantum tomograms, which can be measured in experiments as the prime objects identified with the quantum state of an arbitrary system. The qubit or spin-1/2 state, within the framework of the tomographic probability representation, is identified with the set of three probability distributions of spin projections on three perpendicular directions in the space. This description of the qubit state was studied and illustrated by the triangle geometry of the system, using the so-called Malevich square representation [16] known also as quantum suprematism approach (after the Russian painter Kazmir Malevich (1879–1935), founder of suprematism, an art movement started around 1913 focused on basic geometric figures). Such a geometric representation provides the picture of the qubit state in terms of three squares on the plane obtained through an invertible map of the points in the Bloch sphere onto the probability distributions. This approach has been extended for qutrit states [17,18,19] and, in principle, was generalized to qudit states. An important role of symmetries and group representations, in particular, for spin states was reviewed in [20]. Within the framework of the geometric formulation of quantum mechanics [20,21], an explicit construction of the Fisher–Rao tomographic metric for qubit and qutrit density matrices is established in a quorum of reference frames [22,23]. In addition, using the same approach, the volume of two-qubit states which have maximal random subsystems (where the reduced density matrices ${\hat{\rho }}_{1,2}=\hat{I}/2$), has been calculated in [24] as a function of the purity of the composite system.

Quantum computers manipulate qubits by operations based on Pauli matrices; we elaborate in this work the decomposition of qutrit states into qubit states and hope that the proposed decomposition will also allow the manipulation of qutrits and, in general, of qudits in quantum computing algorithms. An example of the mapping of oscillator creation and annihilation operators onto qubits using the Jordan–Schwinger map [25,26] and manipulation of the qubits in context of information technologies has recently been given in [27].

The aim of this paper is to study, within the probability representation of quantum states [13,14,15,16,17,18,19,28,29,30] reviewed in [31], the triangle geometry, separability, and entanglement of a composite system of two qubits in specific states. In addition, we elaborate the description of the state quantumness by finding new bounds for qubit and qutrit state characteristics presented in terms of square areas given by probability distributions associated with the triangle geometry of their states. It is worth noting that the classical probability distributions and their interference were discussed within the framework of the state vectors in Hilbert space by Khrennikov [32,33,34,35]. Here, the interference is a feature of multi-contextuality. This is not only a problem of classical versus quantum probability, but also quantum versus general contextual probability. The superposition principle for spin-1/2 state vectors was presented in explicit form as the nonlinear superposition of the classical probability distributions determining the qubit states in [19,36,37]. This superposition was illustrated geometrically in the quantum suprematism approach as a superposition of squares. The approach called the suprematism in art is described in [38]. It is worth noting that a methodological relation of the Malevich black square with effectiveness for experimental tools in physics was mentioned in [39].

The system of two qubits can be realized as a system of two two-level atoms; this system has four levels. Specific states of the four-level system are the states where either one level or two levels of the four are not occupied. It means that some states from the set of possible states are inaccessible. We discuss the properties of such states for two-qubit systems. Thus, we study, within the probability representation, the triangle geometry and separability of the specific states of two qubits. This is done by considering that one or two of the composite two-qubit states are not available, which yields to concurrences depending only on two probability distributions of dichotomous random variables. Note that, when there is only one inaccessible state, an additional nonlinear mapping suggested in [18] needs to be applied to determine the geometric picture of the states in terms of three triads of squares. The Peres–Horodecki criterion [40,41] is used to establish the separability or entanglement properties of the two-qubit states.

We point out the following aspects of our approach. The entanglement in a two-qubit system is completely a quantum phenomenon. In view of this fact, it seems to be necessary to use for its description mandatory ingredients such as Hilbert spaces, vectors in the Hilbert space, and density operators acting in the space. As we demonstrate, and it is our goal, it is possible to describe this quantum phenomenon making the identification of qubit states with fair classical-like measurable probabilities. Our conjecture is that other completely quantum phenomena in some other systems such as quantum correlations (e.g., Bell correlations) can also be formally described using the states identification with probability distributions.

This paper is organized as follows.

In Section 2, a short review of the qubit and qutrit state probabilistic description given in the quantum suprematism geometric representation is presented. In Section 3, two-qubit states both separable and entangled are considered in the probability representation when there are one or two inaccessible states. Section 4 presents an example in which the inequalities over the square areas and over the sum of areas lead to conditions which can be used for controlling measurement processes. Conclusions and perspectives are presented in Section 5.

## 2. Qubit and Qutrit States in Quantum Geometric Representation

In this section, we review how the Bloch sphere geometry of qubit states is mapped onto a triangle geometry of qubit and qutrit states. The construction of the map is described in terms of the measurements of probabilities along the quorum of reference frames [16,17,18].

### 2.1. Qubit Case

We start with a qubit density matrix $\hat{\rho }={\hat{\rho }}^{†}$, $\mathrm{Tr}(\hat{\rho })=1$ satisfying the nonnegativity condition of its eigenvalues, i.e.,

(1) $$\hat{\rho }=\left(\begin{array}{cc} \rho_{11} & \rho_{12}\\ \rho_{21} & \rho_{22} \end{array}\right)\;,\;\rho_{21}=\rho_{12}^{*}\;,\;\rho_{11}+\rho_{22}=1\;,$$

and

(2) $$\rho_{11}\;\rho_{22}-\rho_{12}\;\rho_{21}\geq 0\;.$$

The matrix elements $\rho_{jk}$; j,k=1,2 may be constructed in terms of three probability distributions $P_{1}=\left(p_{1},1-p_{1}\right)$, $P_{2}=\left(p_{2},1-p_{2}\right)$, and $P_{3}=\left(p_{3},1-p_{3}\right)$, where $0\leq p_{k}\leq 1$; k=1,2,3 are probabilities of spin-1/2 projections m=±1/2 along the axes x,y,z, respectively. Each probability is related to the expectation values of the projectors

(3) $${\hat{\rho }}_{1}=\frac{1}{2}\left(\begin{array}{cc} 1 & 1\\ 1 & 1 \end{array}\right),\;{\hat{\rho }}_{2}=\frac{1}{2}\left(\begin{array}{cc} 1 & -i\\ i & 1 \end{array}\right),\;{\hat{\rho }}_{3}=\left(\begin{array}{cc} 1 & 0\\ 0 & 0 \end{array}\right)\;,$$

defining the probabilities $\mathrm{Tr}\left(\hat{\rho }{\hat{\rho }}_{k}\right)=p_{k}$ which can be measured experimentally. These measurements allow reconstructing Equation (1) in the form

(4) $$\hat{\rho }=\left(\begin{array}{cc} p_{3} & p_{1}-1/2-i\left(p_{2}-1/2\right)\\ p_{1}-1/2+i\left(p_{2}-1/2\right) & 1-p_{3} \end{array}\right).$$

Note that $p_{1}$, $p_{2}$, and $p_{3}$ are classical probabilities of measuring the projection of angular momentum m=1/2 in three different reference frames. We point out that, for a system of three independent classical coins, its statistics are also described by the same three probabilities.

**Figure 1:**
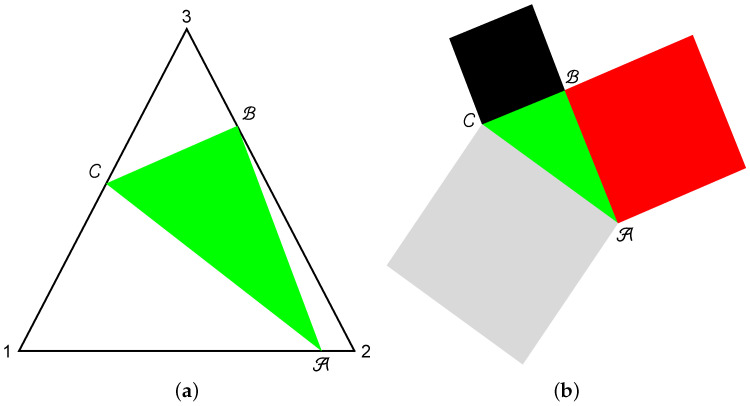
(**a**) Triangle representation of the qubit state by three points along the perimeter of an equilateral triangle of side length $\sqrt{2}$; and (**b**) Malevich’s squares associated to the state.

A state with the density matrix ${\hat{\rho }}_{k}$, as described above, has spin projections m=±1/2 on the three perpendicular directions x,y,z. This means that the state $\hat{\rho }$ is identified with three probabilities $p_{1}$, $p_{2}$, and $p_{3}$. The nonnegativity of the density matrix $\hat{\rho }\geq 0$ provides the condition

(5) $${\left(p_{1}-1/2\right)}^{2}+{\left(p_{2}-1/2\right)}^{2}+{\left(p_{3}-1/2\right)}^{2}\leq 1/4\;,$$

i.e., there exist quantum correlations between the spin projections on the perpendicular directions x,y,z. In contrast, for three classical coins described as the probability vectors $P_{1}$, $P_{2}$, and $P_{3}$, there are no constraints (Equation (5)). The endpoints of the probability vectors $P_{k}$ with components $p_{k}$ and $1-p_{k}$ are situated along 1-simplexes, which form the hypotenuse of rectangular triangles of side 1. If one connects the hypotenuses, one can obtain an equilateral triangle with side length $\sqrt{2}$ (see [16]). Then, the state of a qubit can be represented by three points along the triangle sides, as shown in Figure 1; some new entropic inequalities were obtained for qubit systems in [42].

A, B, and C show the endpoints of vectors $P_{1}$, $P_{2}$, and $P_{3}$ on the simplexes. The side lengths $l_{k}$, k=1,2,3 of the triangle ▵(ABC) can be expressed in terms of probabilities as follows:

(6) $$l_{k}={\left(2p_{k}^{2}+2p_{k+1}^{2}+2p_{k}p_{k+1}-4p_{k}-2p_{k+1}+2\right)}^{1/2}\;.$$

From these, one can define three squares with sides $l_{1}$, $l_{2}$, and $l_{3}$. The triad of squares illustrates the qubit density matrix, and it has a one-to-one correspondence with the Bloch parameters of the state

(7) $$x=2\;p_{1}-1\;,\;y=2\;p_{2}-1\;,\;z=2\;p_{3}-1\;.$$

The linear relation between the probabilities and the Bloch vector parameters, together with the condition in Equation (5), allows an analogous construction to the Bloch sphere with center at $p_{k}=1/2$; k=1,2,3 and radius 1/2. In this representation, the most mixed state with density operator $\hat{\rho }=I/2$ is located at the center of the sphere, and one can find the pure states on the surface.

The sum of the square areas is given in terms of the triangle lengths as $S=l_{1}^{2}+l_{2}^{2}+l_{3}^{2}$ and explicitly in terms of the probabilities as

(8) $$S\left(p_{1},p_{2},p_{3}\right)=2\left(2{p_{1}}^{2}+3\left(1-p_{1}-p_{2}-p_{3}\right)+p_{1}p_{2}+p_{1}p_{3}+2{p_{2}}^{2}+p_{2}p_{3}+2{p_{3}}^{2}\right).$$

The difference with the classical treatment with three coins is that the uncertainty relation in Equation (5) is not imposed. In this classical case, the sum of the square areas satisfies the inequality

(9) $$3/2\leq S_{c}\leq 6\;,$$

where the lower bound corresponds to the probabilities $p_{1}=p_{2}=p_{3}=1/2$ and the upper limit, to $p_{1}=p_{2}=p_{3}=1$.

For the quantum case of the qubit state, one has to consider the constraint in Equation (5). For pure states, i.e., when the equality is satisfied in the uncertainty relation in Equation (5), the sum of the square areas takes local maxima with value $S_{q}=9/4$ and two global maxima with $S_{q}=3$. The lower bound, $S_{q}=3/2$, is given by the maximum mixed states. Therefore, in the quantum case, the sum of the square areas satisfies

(10) $$3/2\leq S_{q}\leq 3\;.$$

For $S_{q}=3$, the triangle ABC is equilateral with the side length equal to 1, and for $S_{q}=3/2$, the equilateral triangle ABC has the side length equal to $\sqrt{2}/2$.

**Figure 2:**
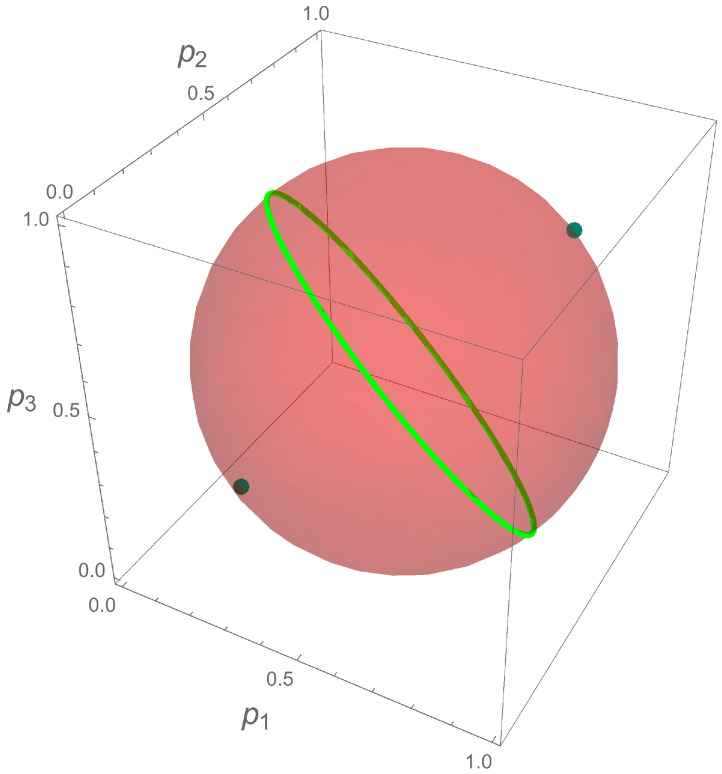
Geometric interpretation of a qubit in the probability representation. The (red) sphere is centered at the maximum mixed state and has radius 1/2. The great circle is associated to pure states, where $S_{q}=9/4$, and the dots are pure states, where $S_{q}=3$.

In Figure 2, we show the geometric interpretation of the qubit state in the probability representation, together with the pure states that maximize the sum of the triad areas $S_{q}$ in the quantum case. The great circle determined by the points

(11) $$p_{2}=\frac{1}{4}\left(3-2p_{1}+\sqrt{-1+12\;p_{1}-12\;p_{1}^{2}}\right),\;p_{3}=\frac{1}{4}\left(3-2p_{1}-\sqrt{-1+12p_{1}-12p_{1}^{2}}\right),$$

where $\left(3-\sqrt{6}\right)/6\leq p_{1}\leq \left(3+\sqrt{6}\right)/6$, corresponds to local maxima. The absolute maxima are reached at the probability vectors

(12) $$\left(p_{1},\;p_{2},\;p_{3}\right)=\left(\frac{1}{6}\left(3-\sqrt{3}\right),\;\frac{1}{6}\left(3-\sqrt{3}\right),\;\frac{1}{6}\left(3-\sqrt{3}\right)\right),\;\;\left(\frac{1}{6}\left(3+\sqrt{3}\right),\;\frac{1}{6}\left(3+\sqrt{3}\right),\;\frac{1}{6}\left(3+\sqrt{3}\right)\right)\;.$$

In addition to the areas, the linear entropy of the system can be calculated using the relation

(13) $$S_{L}=2\sum_{j=1}^{3}p_{j}\left(1-p_{j}\right)-1\;.$$

It is important to note that, if $p_{j}$ represents the standard probability distribution corresponding to a dichotomous random variable (e.g., a coin), the terms $\eta_{j}=p_{j}\left(1-p_{j}\right)$ measure the fairness of the system. If the dichotomous variable has the same probability for both categories $p_{j}=1-p_{j}=1/2$, then $\eta_{j}=1/4$ constitutes the maximum fairness situation. In the opposite case, when one of the categories of the dichotomous variable is not possible, the fairness has a minimum $\eta_{j}=0$.

One can see that, for maximum fairness, the qubit state corresponds to the most mixed state $\hat{\rho }=I/2$ and has a linear entropy $S_{L}=1/2$. When one has minimum fairness, there exist two possibilities: $p_{j}=0$ and $p_{j}=1$. At any of those values, the linear entropy has a value of −1 which is not physical, so one can conclude that the probabilities $p_{j}$ cannot be zero at the same time, nor can they all be equal to 1 or any combination of 0 and 1, in order to represent the qubit state. As can be seen in Figure 2, those points are located outside the permitted sphere given by Equation (5).

In addition, the linear entropy of the system is proportional to the sum of the squared lengths of the triangles $T_{1}=▵\left(AB2\right)$, $T_{2}=▵\left(BC3\right)$, and $T_{3}=▵\left(AC1\right)$, i.e., $\sum_{j=1}^{3}2\left({\left(1-p_{j}\right)}^{2}+p_{j+1}^{2}\right)+l_{j}^{2}$, minus the squared lengths of the triangle $T_{4}=▵\left(ABC\right)$, i.e., $\sum_{j=1}^{3}l_{j}^{2}$; explicitly,

(14) $$S_{L}=2-\sum_{j=1}^{3}\left[{\left(1-p_{j}\right)}^{2}+p_{j+1}^{2}\right]\;,$$

where $p_{4}=p_{1}$. Note that Equations (13) and (14) are equivalent.

### 2.2. Qutrit Case

The probabilistic representation of the qubit state can also be extended to higher dimensions. We consider the example of the qutrit state. The density matrix of the qutrit state

(15) $${\hat{\rho }}_{3}=\left(\begin{array}{ccc} \rho_{11} & \rho_{12} & \rho_{13}\\ \rho_{21} & \rho_{22} & \rho_{23}\\ \rho_{31} & \rho_{32} & \rho_{33} \end{array}\right)\;,$$

can be described using the eight generators of the su(3) algebra represented by the Gell–Mann matrices [43] ${\hat{\lambda }}_{1},\ldots ,{\hat{\lambda }}_{8}$, i.e.,

$${\hat{\rho }}_{3}=\frac{1}{3}\;\hat{I}+\frac{1}{2}\;\sum_{j=1}^{8}a_{j}{\hat{\lambda }}_{j}\;,$$

where $a_{j}\in R$ are the entries of the generalized Bloch vector. Amongst the Gell–Mann matrices, there exist three sets of operators which form su(2) algebras, viz., $\left\{{\hat{\lambda }}_{1},{\hat{\lambda }}_{2},{\hat{\lambda }}_{3}\right\}$, $\left\{{\hat{\lambda }}_{4},{\hat{\lambda }}_{5},\left({\hat{\lambda }}_{3}+\sqrt{3}\;{\hat{\lambda }}_{8}\right)/2\right\}$, and $\left\{{\hat{\lambda }}_{6},{\hat{\lambda }}_{7},\left(-{\hat{\lambda }}_{3}+\sqrt{3}\;{\hat{\lambda }}_{8}\right)/2\right\}$. Given this property, one can think of a possible definition of qubit states using these three sets of operators. An algorithmic procedure to define qubit states is the following: The matrix ${\hat{\rho }}_{3}$ is first extended to two 4 × 4 density matrices, where one row and one column are equal to zero, as follows:

$${\hat{\rho }}_{1}=\left(\begin{array}{cc} {\hat{\rho }}_{3} & 0\\ 0 & 0 \end{array}\right)\;,\;{\hat{\rho }}_{2}=\left(\begin{array}{cc} 0 & 0\\ 0 & {\hat{\rho }}_{3} \end{array}\right)\;.$$

**Figure 3:**
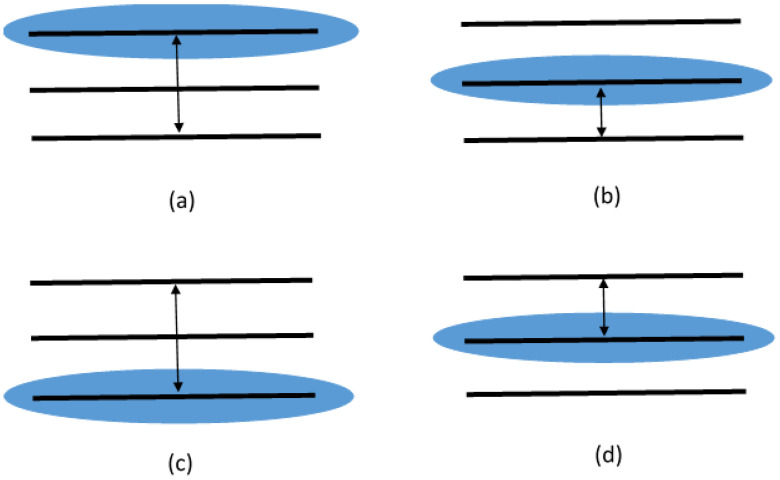
Schematic representation of qubits defined by a generic three-level system given by the density matrices: (**a**) ${\hat{\rho }}^{\left(A\right)}$; (**b**) ${\hat{\rho }}^{\left(B\right)}$; (**c**) ${\hat{\rho }}^{\left(C\right)}$; and (**d**) ${\hat{\rho }}^{\left(D\right)}$. In all cases, the occupation number of the states in blue define the diagonal terms, while the arrows denote the transitions which define the off-diagonal terms of the qubits.

Interpreting the resulting matrices as density operators for two qubit systems, we make use of the partial trace operation to define four matrices that must be positive semidefinite ${\hat{\rho }}^{\left(A\right)}$, ${\hat{\rho }}^{\left(B\right)}$, ${\hat{\rho }}^{\left(C\right)}$, and ${\hat{\rho }}^{\left(D\right)}$, which are not independent

(16) $$\begin{array}{c} {\hat{\rho }}^{\left(A\right)}=\left(\begin{array}{cc} 1-\rho_{33} & \rho_{13}\\ \rho_{31} & \rho_{33} \end{array}\right)\;,\;{\hat{\rho }}^{\left(B\right)}=\left(\begin{array}{cc} 1-\rho_{22} & \rho_{12}\\ \rho_{21} & \rho_{22} \end{array}\right)\;,\\ {\hat{\rho }}^{\left(C\right)}=\left(\begin{array}{cc} \rho_{11} & \rho_{13}\\ \rho_{31} & 1-\rho_{11} \end{array}\right)\;,\;{\hat{\rho }}^{\left(D\right)}=\left(\begin{array}{cc} \rho_{22} & \rho_{23}\\ \rho_{32} & 1-\rho_{22} \end{array}\right)\;; \end{array}$$

in Figure 3, it is shown that associated to any of the qubit density matrices in Equation (16) is a three-level system. In each case, the population of one of the levels with the transition probability to another level determine different qubits. It can be seen that the off-diagonal components of the matrices in Equation (16) are naturally arranged in the sets given by the su(2) algebras, i.e., $A:\{a_{4},a_{5}\}$, $B:\{a_{1},a_{2}\}$, $C:\{a_{4},a_{5}\}$, and $D:\{a_{6},a_{7}\}$. Therefore, each one of these density matrices can be decomposed in terms of three probabilities as described in Equation (4). Choosing the independent qubits as ${\hat{\rho }}^{\left(A\right)}$, ${\hat{\rho }}^{\left(B\right)}$, ${\hat{\rho }}^{\left(D\right)}$, one can retrieve the original 3 × 3 density matrix in the form

(17) $${\hat{\rho }}_{3}=\left(\begin{array}{ccc} p_{3}^{\left(A\right)}+p_{3}^{\left(B\right)}-1 & B & A\\ B^{*} & 1-p_{3}^{\left(B\right)} & D\\ A^{*} & D^{*} & 1-p_{3}^{\left(A\right)} \end{array}\right)\;,$$

where $A=p_{1}^{\left(A\right)}-1/2-i\left(p_{2}^{\left(A\right)}-1/2\right)$, $B=p_{1}^{\left(B\right)}-1/2-i\left(p_{2}^{\left(B\right)}-1/2\right)$, and $D=p_{1}^{\left(D\right)}-1/2-i\left(p_{2}^{\left(D\right)}-1/2\right)$; here, the numbers $p_{1,2,3}^{\left(A),(B),(D\right)}$ are probabilities satisfying the inequality in Equation (5). It is worth mentioning that qubits ${\hat{\rho }}^{\left(B\right)}$, ${\hat{\rho }}^{\left(C\right)}$, and ${\hat{\rho }}^{\left(D\right)}$ can also be used to describe the system, as shown below.

The qubit probabilities can be obtained in terms of the tomographic probabilities used for the state reconstruction. It is known that, to reconstruct the qutrit state, one needs to measure the probabilities corresponding to the spin projections m=0,1 on the *z* axis in four different reference frames. Each of these frames constitute a general rotation of the density matrix acting by the operator $\hat{U}=\prod_{j=1}^{8}\exp\left(i\theta_{j}{\hat{\lambda }}_{j}\right)$ on the original state ${\hat{U}}^{†}{\hat{\rho }}_{3}\hat{U}$.

As in the qubit case, the linear entropy of the system can be obtained as

(18) $$S_{L}=2\left(\sum_{j=A,B,D}\sum_{k=1}^{3}p_{k}^{\left(j\right)}\left(1-p_{k}^{\left(j\right)}\right)+p_{3}^{\left(A\right)}\left(1-p_{3}^{\left(B\right)}\right)+p_{3}^{(B)2}\right)-5\;,$$

with $p_{3}^{\left(D\right)}=1-p_{3}^{\left(B\right)}$. Even though the expression is similar, one can see that, in addition to the fairness terms for each probability $\eta_{jk}=p_{k}^{\left(j\right)}\left(1-p_{k}^{\left(j\right)}\right)$, we also have the joint probability distribution $p_{3}^{\left(A\right)}\left(1-p_{3}^{\left(B\right)}\right)$, and the probability $p_{3}^{\left(B\right)}$. It can be shown that $S_{L}^{\left(A\right)}+S_{L}^{\left(B\right)}+S_{L}^{\left(D\right)}=2\sum_{j=A,B,C}\sum_{k=1}^{3}p_{k}^{\left(j\right)}\left(1-p_{k}^{\left(j\right)}\right)-3$, so the linear entropy is expressed as

(19) $$S_{L}=\sum_{j=A,B,D}S_{L}^{\left(j\right)}-2\left(1-p_{3}^{\left(B\right)}\right)\left(1+p_{3}^{\left(B\right)}-p_{3}^{\left(A\right)}\right)\;,$$

which can be obtained geometrically, in view of the property of the entropy for the three qubits *A*, *B*, and *D* in terms of the squared lengths of the triad squares, as discussed previously. It is important to note that, in general, the sum of the linear entropies for qubits is larger than the linear entropy of the qutrit, i.e., $S_{L}\leq \sum_{j}S_{L}^{\left(j\right)}$.

Given the nonnegativity of the qutrit density matrix, there exist correlations between its matrix components, i.e., if a change in the system is done, these components must change in a way to guaranty the hermiticity and nonnegativity of the state. Even if we might be able to change a single matrix element of the state, a change in all the others would take place after. These correlations also imply a correlation between the component qubits defined above. For these reasons, one can think of Equation (19) as a way to measure correlations between different components of the qutrit state, that is between different qubits.

Next, we determine the bounds associated to the sum of the square areas for the qutrit in the B,C,D qubit representation,

(20) $$S=S\left(p_{1}^{\left(B\right)},\;p_{2}^{\left(B\right)},\;p_{3}^{\left(B\right)}\right)+S\left(p_{1}^{\left(C\right)},\;p_{2}^{\left(C\right)},\;p_{3}^{\left(C\right)}\right)+S\left(p_{1}^{\left(D\right)},\;p_{2}^{\left(D\right)},\;1-p_{3}^{\left(B\right)}\right)\;.$$

**Figure 4:**
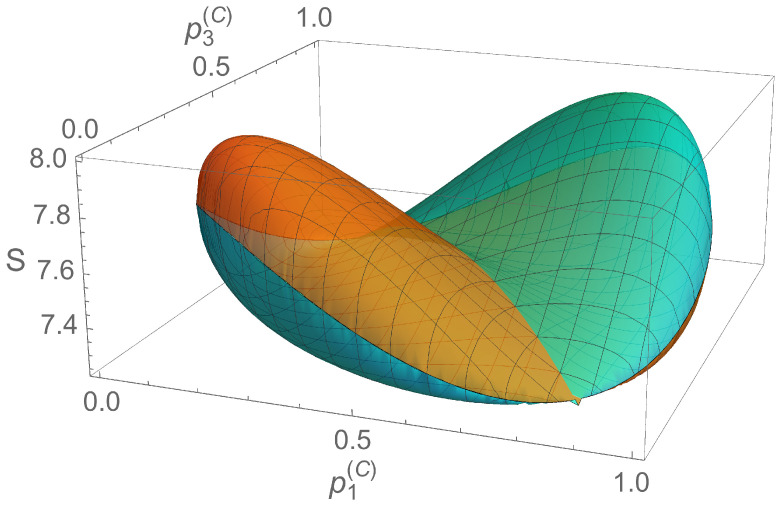
The pure qubit representation of the sum of the square areas in the probability space of $\left(p_{1}^{\left(C\right)},p_{3}^{\left(C\right)}\right)$. It corresponds to pure qutrit states. Each color denotes independent solutions.

We demonstrated that the qutrit density matrix can be written in terms of eight probabilities establishing a three-qubit representation. By requiring the purity of the qutrit and the fact that qubits correspond also to pure states, one can reduce the number of free probabilities to $p_{1}^{\left(C\right)}$ and $p_{3}^{\left(C\right)}$. The minimum value of the sum of the square areas is obtained when all the probabilities take the value 1/2, which corresponds to a diagonal density matrix for the qutrit, diag(1/2,1/2,0). The maximum value for the qutrit in the pure qubit representation reads S=8, while the minimum is 29/4. The region of $\left(p_{1}^{\left(C\right)},p_{3}^{\left(C\right)}\right)$ formed with pure qubit states is the surface shown in Figure 4. The extreme bounds are given by

(21) $$\frac{9}{2}\leq S≲8.1565\;,$$

where the upper bound is associated with the pure state and the probabilities $p_{1}^{\left(B\right)}\approx 0.5733$, $p_{2}^{\left(B\right)}\approx 0.5207$, $p_{3}^{\left(B\right)}\approx 0.9716$, $p_{1}^{\left(C\right)}\approx 0.2379$, $p_{2}^{\left(C\right)}\approx 0.2031$, $p_{3}^{\left(C\right)}\approx 0.2044$, $p_{1}^{\left(D\right)}\approx 0.3760$, and $p_{2}^{\left(D\right)}\approx 0.4200$. The discussed values are obtained using numerical calculations. It can be seen that these values for the probabilities imply that the pairs of qubits ${\hat{\rho }}^{\left(A\right)}$, ${\hat{\rho }}^{\left(B\right)}$ and ${\hat{\rho }}^{\left(C\right)}$, ${\hat{\rho }}^{\left(D\right)}$ have the same purity, and that it is close to the unity. States reaching this upper bound are shown in the Appendix A. The lower bound corresponds to $p_{j}^{\left(B),(C),(D\right)}=1/2$, with j=1,2,3.

We note that different parameterizations do not lead to the same maxima of the areas. For example, the A,B,D parameterization allows for greater purity of qubits, thus yielding a lower total sum of areas. If one requires the purity of the qutrit to be equal to 1, and equal purities for ${\hat{\rho }}^{\left(A\right)}$ and ${\hat{\rho }}^{\left(B\right)}$, and for ${\hat{\rho }}^{\left(C\right)}$ and ${\hat{\rho }}^{\left(D\right)}$, this yields a maximum value of S≈8.095.

## 3. Separability Properties of the Two-Qubit Composite Systems

Given the density matrix of two qubits in the form $\rho_{m_{1},m_{2},m_{1}^{\prime },m_{2}^{\prime }}$, ($m_{1},m_{2},m_{1}^{\prime },m_{2}^{\prime }=\pm 1/2$), i.e.,

(22) $$\hat{\rho }\left(1,2\right)=\left(\begin{array}{cccc} \rho_{\frac{1}{2},\frac{1}{2},\frac{1}{2},\frac{1}{2}} & \rho_{\frac{1}{2},\frac{1}{2},\frac{1}{2},-\frac{1}{2}} & \rho_{\frac{1}{2},\frac{1}{2},-\frac{1}{2},\frac{1}{2}} & \rho_{\frac{1}{2},\frac{1}{2},-\frac{1}{2},-\frac{1}{2}}\\ \rho_{\frac{1}{2},-\frac{1}{2},\frac{1}{2},\frac{1}{2}} & \rho_{\frac{1}{2},-\frac{1}{2},\frac{1}{2},-\frac{1}{2}} & \rho_{\frac{1}{2},-\frac{1}{2},-\frac{1}{2},\frac{1}{2}} & \rho_{\frac{1}{2},-\frac{1}{2},-\frac{1}{2},-\frac{1}{2}}\\ \rho_{-\frac{1}{2},\frac{1}{2},\frac{1}{2},\frac{1}{2}} & \rho_{-\frac{1}{2},\frac{1}{2},\frac{1}{2},-\frac{1}{2}} & \rho_{-\frac{1}{2},\frac{1}{2},-\frac{1}{2},\frac{1}{2}} & \rho_{-\frac{1}{2},\frac{1}{2},-\frac{1}{2},-\frac{1}{2}}\\ \rho_{-\frac{1}{2},-\frac{1}{2},\frac{1}{2},\frac{1}{2}} & \rho_{-\frac{1}{2},-\frac{1}{2},\frac{1}{2},-\frac{1}{2}} & \rho_{-\frac{1}{2},-\frac{1}{2},-\frac{1}{2},\frac{1}{2}} & \rho_{-\frac{1}{2},-\frac{1}{2},-\frac{1}{2},-\frac{1}{2}} \end{array}\right)\;,$$

we consider two different situations for the two-qubit systems. The first one where two states are not available or forbidden, while in the second case only one is inaccessible. We analyze these different possibilities below.

### 3.1. Two Inaccessible States

A two-qubit density matrix (Equation (22)), in which two of the states (with $m_{1},m_{2}=1/2,1/2$ and −1/2,−1/2) are inaccessible, can be expressed as

(23) $$\hat{\rho }\left(1,2\right)=\left(\begin{array}{cccc} 0 & 0 & 0 & 0\\ 0 & \rho_{11} & \rho_{12} & 0\\ 0 & \rho_{21} & \rho_{22} & 0\\ 0 & 0 & 0 & 0 \end{array}\right)\;.$$

This state can be related to an equilibrium density operator $\hat{\rho }=e^{-\hat{H}/T}/\mathrm{Tr}\left(e^{-\hat{H}/T}\right)$, where the Hamiltonian has very large first and fourth eigenvalues in comparison with the other two, so that the transitions to the corresponding eigenstates are forbidden. Since the qubit density matrix is expressed in terms of the probabilities $p_{1}$, $p_{2}$, and $p_{3}$, Equation (23) can be written as

(24) $$\hat{\rho }\left(1,2\right)=\left(\begin{array}{cccc} 0 & 0 & 0 & 0\\ 0 & p_{3} & p_{1}-1/2-i\left(p_{2}-1/2\right) & 0\\ 0 & p_{1}-1/2+i\left(p_{2}-1/2\right) & 1-p_{3} & 0\\ 0 & 0 & 0 & 0 \end{array}\right)\;.$$

Next, we present a quantification of the entanglement by means of the negativity [44] and concurrence [45,46] concepts. The negativity is defined by the sum of the absolute values of the negative eigenvalues of the ppt density matrix ${\hat{\rho }}^{PT}$, that is, $N\left(\hat{\rho }\right)=\sum_{k}\left|{\lambda_{k}}^{\left(-\right)}\right|$. Thus, one constructs the partial transpose density matrix in the probability representation, which has eigenvalues $\lambda_{1}=p_{3}$, $\lambda_{2}=1-p_{3}$, $\lambda_{3}=\sqrt{{\left(p_{1}-1/2\right)}^{2}+{\left(p_{2}-1/2\right)}^{2}}$, $\lambda_{4}=-\lambda_{3}$. These probabilities satisfy Equation (5), hence the negativity of the system is

(25) $$N\left(\hat{\rho }\right)=\sqrt{{\left(p_{1}-1/2\right)}^{2}+{\left(p_{2}-1/2\right)}^{2}}\;,$$

and we immediately see that, for special values of $p_{1}=p_{2}=1/2$, the two-qubit state is separable. For all the other values of the probabilities, the state is entangled.

**Figure 5:**
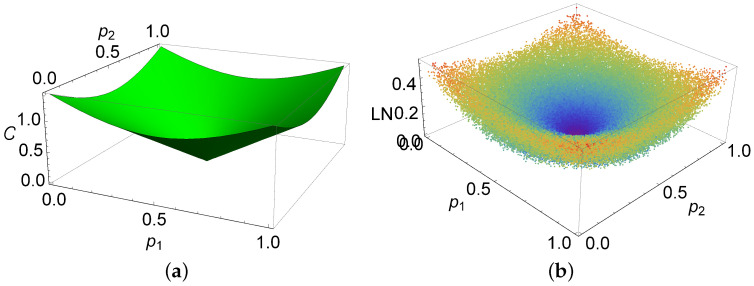
(**a**) Quantum concurrence for $\hat{\rho }\left(1,2\right)$; and (**b**) the numeric logarithmic negativity for the density matrix ${\hat{\rho }}_{1}\left(1,2\right)$ in terms of the corresponding probabilities $p_{1}$ and $p_{2}$.

We obtain the concurrence of the system by calculating the square root of the eigenvalues of the matrix $\hat{\rho }{\hat{\rho }}^{\prime }$, where ${\hat{\rho }}^{\prime }=\left({\hat{\sigma }}_{y}⊗{\hat{\sigma }}_{y}\right){\hat{\rho }}^{*}\left({\hat{\sigma }}_{y}⊗{\hat{\sigma }}_{y}\right)$, ${\hat{\rho }}^{*}$ is the complex conjugate of $\hat{\rho }$, and with ${\hat{\sigma }}_{y}$ being the Pauli matrix. The square root of the eigenvalues of such a matrix in descending order ($\eta_{1}$, $\eta_{2}$, $\eta_{3}$, and $\eta_{4}$) defines the concurrence $C=\max\left(0,\eta_{1}-\eta_{2}-\eta_{3}-\eta_{4}\right)$. Given the state of Equation (24), these are

$$\eta_{1,2}=\sqrt{p_{3}\left(1-p_{3}\right)}\pm \sqrt{{\left(p_{1}-1/2\right)}^{2}+{\left(p_{2}-1/2\right)}^{2}}\;,\;\eta_{3,4}=0\;;$$

thus, the concurrence of the state is

(26) $$C=2\sqrt{{\left(p_{1}-1/2\right)}^{2}+{\left(p_{2}-1/2\right)}^{2}}=2\left|\hat{\rho }{\left(1,2\right)}_{23}\right|=2N\left(\hat{\rho }\right)\;;$$

it is shown in Figure 5a. Here, we see that the concurrence is zero when $p_{1}=p_{2}=1/2$, i.e., when the state is diagonal, and has a maximum value when both probabilities are equal to one of the extreme values, zero or one; this corresponds to the different states, where $\hat{\rho }{\left(1,2\right)}_{2,3}$ is either (−1±i)/2 or (1±i)/2, and the inequality $p_{3}\left(1-p_{3}\right)\leq 3/4$ is satisfied.

We can also analyze the separability of the states in terms of the square areas. This can be done by taking such four matrix elements that are different from zero as a qubit. In the case where the system is separable, $N(\hat{\rho })=0$; $p_{1}=p_{2}=1/2$, one has from Equation (5) that the value of the other probability is unrestricted $0\leq p_{3}\leq 1$. However, the sum of the square areas $S=p_{3}\left(4p_{3}-5\right)+3$ can take values 3/2≤S≤5/2, while if the system is entangled the probabilities ${\left(p_{1}-1/2\right)}^{2}+{\left(p_{2}-1/2\right)}^{2}=N^{2}\left(\hat{\rho }\right)$ are located within a circle of radius equal to the negativity of the system, and we should have $1/2\left(1-\sqrt{1-4\;N^{2}\left(\hat{\rho }\right)}\right)\leq p_{3}\leq 1/2\left(1+\sqrt{1-4\;N^{2}\left(\hat{\rho }\right)}\right)$. Since the negativity takes a value between 0 and 1/2, we have $0\leq p_{3}\leq 1/2$. From these arguments, one can see that the sum of the square areas can take any value between 3/2 and 3. The interval (5/2,3] for *S* provides the possibility for experimental checkup of the entanglement of the system $\hat{\rho }\left(1,2\right)$ in terms of probabilities.

Now, consider the case where the state is given by the density matrix

(27) $$\hat{\rho }\left(1,2\right)=\left(\begin{array}{cccc} p_{3} & 0 & 0 & p_{1}-1/2-i\left(p_{2}-1/2\right)\\ 0 & 0 & 0 & 0\\ 0 & 0 & 0 & 0\\ p_{1}-1/2+i\left(p_{2}-1/2\right) & 0 & 0 & 1-p_{3} \end{array}\right)\;.$$

As in the previous case, the state can be written in the form $\hat{\rho }=e^{-\hat{H}/T}/\mathrm{Tr}\left(e^{-\hat{H}/T}\right)$, where the Hamiltonian has very large second and third eigenvalues compared with the other two.

The eigenvalues of the partial transpose are the same as in the previous example, so the negativity is also given by Equation (25), and the concurrence provides the same result of Equation (26). Hence, one can conclude that there is entanglement for $p_{1,2}\neq 1/2$.

### 3.2. One Inaccessible State

In this case, the density operator can be described by a 3 × 3-matrix inside the general 4 × 4-matrix. To establish its qubit representation, we consider, following [18], density matrices of the form

(28) $${\hat{\rho }}_{1}\left(1,2\right)=\left(\begin{array}{cccc} R_{11} & R_{12} & R_{13} & 0\\ R_{21} & R_{22} & R_{23} & 0\\ R_{31} & R_{32} & R_{33} & 0\\ 0 & 0 & 0 & 0 \end{array}\right)\;,\;{\hat{\rho }}_{2}\left(1,2\right)=\left(\begin{array}{cccc} 0 & 0 & 0 & 0\\ 0 & R_{11} & R_{12} & R_{13}\\ 0 & R_{21} & R_{22} & R_{23}\\ 0 & R_{31} & R_{32} & R_{33} \end{array}\right)\;,$$

where the matrix $\hat{R}$ with elements $R_{jk}$; j,k=1,2,3 is the qutrit density matrix. Since the qutrit is given in the probability representation by Equation (17), the two-qubit system represented by $\hat{\rho }\left(1,2\right)$ can be also expressed in terms of probabilities.

To study the properties of entanglement, we use the Peres–Horodecki criterion and construct the positive partial transpose matrix ${\hat{\rho }}^{PT}\left(1,2\right)$ with the map $T_{2}=I⊗T$, where *T* stands for the transpose operator, which yields to two matrices; one for each matrix in Equation (28),

(29) $${\hat{\rho }}_{1}^{PT}\left(1,2\right)=\left(\begin{array}{cccc} R_{11} & R_{21} & R_{13} & R_{23}\\ R_{12} & R_{22} & 0 & 0\\ R_{31} & 0 & R_{33} & 0\\ R_{32} & 0 & 0 & 0 \end{array}\right)\;,\;{\hat{\rho }}_{2}^{PT}\left(1,2\right)=\left(\begin{array}{cccc} 0 & 0 & 0 & R_{12}\\ 0 & R_{11} & 0 & R_{13}\\ 0 & 0 & R_{22} & R_{32}\\ R_{21} & R_{31} & R_{23} & R_{33} \end{array}\right)\;.$$

The criterion reads: If any of the eigenvalues of the matrices in Equation (29) is negative, then the states described by the matrices in Equation (28) are entangled.

As an example, we consider the state ${\hat{\rho }}_{1}\left(1,2\right)$ of Equation (28), with each one of its elements described by the probabilities as in Equation (17). This time, the square root of the eigenvalues of $\hat{\rho }{\hat{\rho }}^{\prime }$ are $\eta_{1,2}=\sqrt{\left(1-p_{3}^{\left(A\right)}\right)\left(1-p_{3}^{\left(B\right)}\right)}\pm \left|D\right|$ and $\eta_{3,4}=0$. From this, the concurrence is

(30) $$C=2|D|=2\sqrt{{\left(p_{1}^{\left(D\right)}-1/2\right)}^{2}+{\left(p_{2}^{\left(D\right)}-1/2\right)}^{2}}\;,$$

implying entanglement when D≠0$\left(p_{1,2}^{\left(D\right)}\neq 1/2\right)$.

In addition, the separability condition C=0 implies that the sum of the square areas for qubit ${\hat{\rho }}^{\left(D\right)}$ is restricted to values between 3/2 and 5/2. Thus, in the separable case, the value of the sum of the areas of the triads is bounded by the range 9/2≤S≤8. The value of the sum S=8 is attained when $p_{1}^{\left(B\right)}=p_{2}^{\left(B\right)}=p_{1}^{\left(D\right)}=p_{2}^{\left(D\right)}=1/2$, $p_{3}^{\left(B\right)}=1$ and $p_{1}^{\left(C\right)}=p_{2}^{\left(C\right)}=p_{3}^{\left(C\right)}=\left(3+\sqrt{3}\right)/6$.

In the case of ${\hat{\rho }}_{2}$, the square root of the eigenvalues of $\hat{\rho }{\hat{\rho }}^{\prime }$ are $\eta_{1,2}=\sqrt{\left(p_{3}^{\left(A\right)}+p_{3}^{\left(B\right)}-1\right)\left(1-p_{3}^{\left(B\right)}\right)}\pm \left|B\right|$ and $\eta_{3,4}=0$. From these values, the concurrence is calculated to be

(31) $$C=2|B|=2\sqrt{{\left(p_{1}^{\left(B\right)}-1/2\right)}^{2}+{\left(p_{2}^{\left(B\right)}-1/2\right)}^{2}}\;,$$

which means that the system is separable when B=0. In addition, we can notice that the sum of the square areas has the same bounds as in the previous case (9/2≤S≤8).

Finally, we consider the state

(32) $$\hat{\rho }=\left(\begin{array}{cccc} R_{11} & 0 & R_{12} & R_{13}\\ 0 & 0 & 0 & 0\\ R_{21} & 0 & R_{22} & R_{23}\\ R_{31} & 0 & R_{32} & R_{33} \end{array}\right)\;.$$

We found the eigenvalues $\eta_{1,2}=\sqrt{\left(p_{3}^{\left(A\right)}+p_{3}^{\left(B\right)}-1\right)\left(1-p_{3}^{\left(A\right)}\right)}\pm \left|A\right|$, $\eta_{3,4}=0$, and the concurrence of the form

(33) $$C=2|A|=2\sqrt{{\left(p_{1}^{\left(A\right)}-1/2\right)}^{2}+{\left(p_{2}^{\left(A\right)}-1/2\right)}^{2}}\;.$$

It can be shown that the sum of the square areas for the separable case has the bounds $9/2\leq S\leq (57+\sqrt{17})/8$, where the maximum is obtained when $\hat{\rho }$ describes the pure state, the qubits have the same purity $\mathrm{Tr}\left({\hat{\rho }}^{(A)2}\right)=\mathrm{Tr}\left({\hat{\rho }}^{(B)2}\right)$ and $\mathrm{Tr}\left({\hat{\rho }}^{(C)2}\right)=\mathrm{Tr}\left({\hat{\rho }}^{(D)2}\right)$, with one of these purities equal to 1. This can be attained for the probabilities $p_{1}^{\left(B\right)}=p_{2}^{\left(B\right)}=p_{1}^{\left(C\right)}=p_{2}^{\left(C\right)}=1/2$, $p_{3}^{\left(B\right)}=1/2\left(1\pm \sqrt{1/2+3/(2\sqrt{17})}\right)$, $p_{3}^{\left(C\right)}=0$, and $p_{1,2}^{\left(D\right)}=1/2\mp 1/4\sqrt{1-3/\sqrt{17}}$.

The entanglement properties of the physical system described by the density matrix, in which the third row and third column vanish, are analogous to those of ${\hat{\rho }}_{1}\left(1,2\right)$. In this case, all the expressions for the concurrence have the same analytic form as for the two inaccessible states; they are also depicted in Figure 5a. The separability of the systems, when D=0 or B=0 or A=0, can be checked using the partial transpose procedure. In all these cases, the eigenvalues of the partial transpose are equal to the nonnegative eigenvalues of the original density matrix, so the negativity vanishes.

When the system state is not separable, the calculation of the negativity can only be done numerically. In Figure 5b, we illustrate the behavior of the logarithmic negativity $LN\left(\hat{\rho }\right)=\ln\left(2\;N\left(\hat{\rho }\right)+1\right)$ for the system ${\hat{\rho }}_{1}\left(1,2\right)$. We notice that the logarithmic negativity is zero for the values $p_{1}=p_{2}=1/2$, and the state is diagonal. In addition, the logarithmic negativity has a maximum value when both probabilities correspond to an extreme value, zero or one. The probabilities $p_{1}$ and $p_{2}$ at the extremal values of the logarithmic negativity are the same as the ones for the concurrence.

## 4. Example

We now consider the coherent state for spin J=1 (cf., e.g., [47])

$$|\zeta \rangle =\frac{1}{1+{\left|\zeta \right|}^{2}}(|1,-1\rangle +\sqrt{2}\;\zeta |1,0\rangle +\zeta^{2}|1,1\rangle )\;,$$

where ζ is a complex parameter given in terms of the polar and azimuthal angles of the Bloch sphere. This state is interesting in regards to the Einstein-Podolsky-Rosen paradox experiment when taken as a symmetric state of two spin-1/2 particles. The fact that one may determine the separability or entanglement of the two particles by only measuring 2 components of $\hat{J}$ in a series of runs may provide an advantage to experimental setups.

This pure state defines the following qubit probabilities in terms of the mean values of the spin operators ${\hat{J}}_{x}$, ${\hat{J}}_{y}$, and ${\hat{J}}_{z}$

(34) $$\begin{array}{ccc} p_{1}^{\left(A\right)}=\frac{1}{4}\left(2+{\langle {\hat{J}}_{x}\rangle }^{2}-{\langle {\hat{J}}_{y}\rangle }^{2}\right), & p_{2}^{\left(A\right)}=\frac{1}{2}\left(1+\langle {\hat{J}}_{x}\rangle \langle {\hat{J}}_{y}\rangle \right), & p_{3}^{\left(A\right)}=\frac{1}{4}\left(3-\langle {\hat{J}}_{z}\rangle \right)\left(1+\langle {\hat{J}}_{z}\rangle \right)\;,\\ p_{1}^{\left(B\right)}=\frac{1}{4}\left(2+\sqrt{2}\langle {\hat{J}}_{x}\rangle \left(1+\langle {\hat{J}}_{z}\rangle \right)\right), & p_{2}^{\left(B\right)}=\frac{1}{4}\left(2+\sqrt{2}\langle {\hat{J}}_{y}\rangle \left(1+\langle {\hat{J}}_{z}\rangle \right)\right), & p_{3}^{\left(B\right)}=\frac{1}{2}\left(1+{\langle {\hat{J}}_{z}\rangle }^{2}\right)\;,\\ p_{1}^{\left(D\right)}=\frac{1}{4}\left(2+\sqrt{2}\langle {\hat{J}}_{x}\rangle \left(1-\langle {\hat{J}}_{z}\rangle \right)\right), & p_{2}^{\left(D\right)}=\frac{1}{4}\left(2+\sqrt{2}\langle {\hat{J}}_{y}\rangle \left(1-\langle {\hat{J}}_{z}\rangle \right)\right), & p_{3}^{\left(D\right)}=\frac{1}{2}\left(1-\langle {\hat{J}}_{z}\rangle \right)\left(1+\langle {\hat{J}}_{z}\rangle \right)\;, \end{array}$$

via which the classical probabilities can be measured experimentally. Although these expressions depend of the three mean values, the dependence can be reduced to only two by the property ${\langle {\hat{J}}_{x}\rangle }^{2}+{\langle {\hat{J}}_{y}\rangle }^{2}+{\langle {\hat{J}}_{z}\rangle }^{2}=1$. Given this, one can immediately check the constrictions for every one of the qubits (Equation (5)), resulting in $0\leq \frac{1}{8}\left({\langle {\hat{J}}_{z}\rangle }^{4}\mp 2{\langle {\hat{J}}_{z}\rangle }^{3}\pm 2\langle {\hat{J}}_{z}\rangle +1\right)\leq 1/4$, which can be reduced to the standard condition $-1\leq \langle {\hat{J}}_{z}\rangle \leq 1$. Furthermore, the inequalities over the squares areas (Equation (10)) for every one of the three qubits *A*, *B*, and *D*, lead to the expressions

(35) $$\begin{array}{c} \frac{3}{2}\leq \frac{5{\langle {\hat{J}}_{z}\rangle }^{4}}{8}-\frac{5{\langle {\hat{J}}_{z}\rangle }^{3}}{4}+\frac{1}{4}{\langle {\hat{J}}_{z}\rangle }^{2}\left(-2\langle {\hat{J}}_{x}\rangle \langle {\hat{J}}_{y}\rangle +{\langle {\hat{J}}_{y}\rangle }^{2}-1\right)+\frac{1}{4}\langle {\hat{J}}_{z}\rangle \left(2\langle {\hat{J}}_{y}\rangle \left(\langle {\hat{J}}_{x}\rangle -\langle {\hat{J}}_{y}\rangle \right)+5\right)+\\ \;\frac{1}{8}\left(17-2\langle {\hat{J}}_{y}\rangle \left(2\langle {\hat{J}}_{x}\rangle \left({\langle {\hat{J}}_{y}\rangle }^{2}-1\right)+\langle {\hat{J}}_{y}\rangle \right)\right)\leq 3\;,\\ \frac{3}{2}\leq \frac{{\langle {\hat{J}}_{z}\rangle }^{4}}{2}+\frac{1}{4}{\langle {\hat{J}}_{z}\rangle }^{3}\left(\sqrt{2}\langle {\hat{J}}_{x}\rangle +\sqrt{2}\langle {\hat{J}}_{y}\rangle \mp 4\right)+\frac{1}{4}{\langle {\hat{J}}_{z}\rangle }^{2}\left(\langle {\hat{J}}_{x}\rangle \left(\langle {\hat{J}}_{y}\rangle \pm \sqrt{2}\right)\pm \sqrt{2}\langle {\hat{J}}_{y}\rangle \right)\pm \\ \;\langle {\hat{J}}_{z}\rangle \left(\frac{\langle {\hat{J}}_{x}\rangle \langle {\hat{J}}_{y}\rangle }{2}+1\right)+\frac{\langle {\hat{J}}_{x}\rangle \langle {\hat{J}}_{y}\rangle }{4}+2\leq 3\;. \end{array}$$

Again, these inequalities are constrained to $\langle {\hat{J}}_{x}\rangle =\pm \sqrt{1-{\langle {\hat{J}}_{y}\rangle }^{2}-{\langle {\hat{J}}_{z}\rangle }^{2}}$. On the other hand, the sum of the square areas (Equation (20)) define the following inequality:

(36) $$\begin{array}{c} \frac{9}{2}\leq \frac{13{\langle {\hat{J}}_{z}\rangle }^{4}}{8}+{\langle {\hat{J}}_{z}\rangle }^{3}\left(\frac{\langle {\hat{J}}_{x}\rangle }{\sqrt{2}}+\frac{\langle {\hat{J}}_{y}\rangle }{\sqrt{2}}-\frac{5}{4}\right)+\frac{1}{4}\left({\langle {\hat{J}}_{y}\rangle }^{2}-1\right){\langle {\hat{J}}_{z}\rangle }^{2}+\frac{1}{4}\langle {\hat{J}}_{z}\rangle \left(2\langle {\hat{J}}_{y}\rangle \left(\langle {\hat{J}}_{x}\rangle -\langle {\hat{J}}_{y}\rangle \right)+5\right)+\\ \frac{1}{8}\left(49-2\langle {\hat{J}}_{y}\rangle \left(2\langle {\hat{J}}_{x}\rangle \left({\langle {\hat{J}}_{y}\rangle }^{2}-2\right)+\langle {\hat{J}}_{y}\rangle \right)\right)≲8.095\;. \end{array}$$

**Figure 6:**
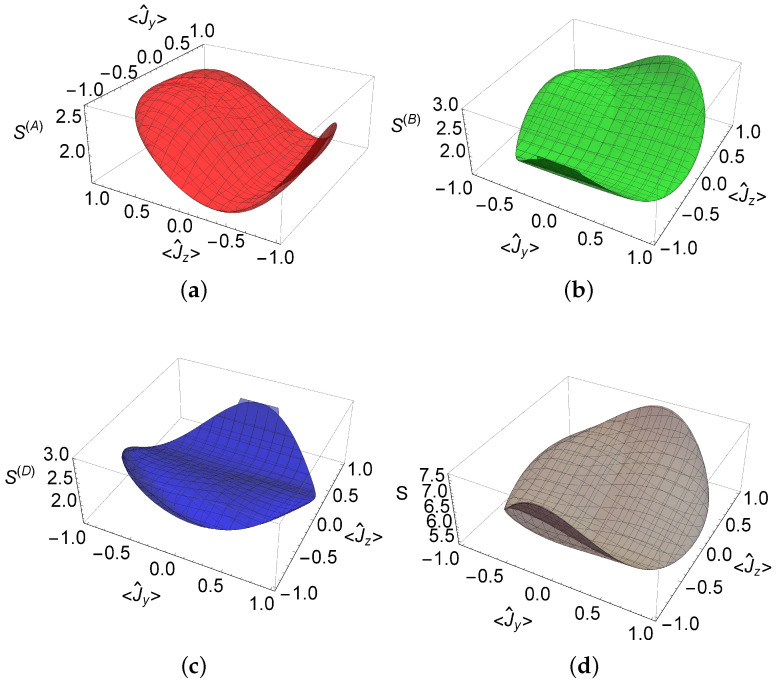
Sum of the square areas for the qubits: (**a**) ${\hat{\rho }}^{\left(A\right)}$; (**b**) ${\hat{\rho }}^{\left(B\right)}$; and (**c**) ${\hat{\rho }}^{\left(D\right)}$. (**d**) Total sum of the areas *S*. All these functions depend of the mean values of the spin operators ${\hat{J}}_{y}$ and ${\hat{J}}_{z}$ of the coherent state |ζ〉.

As the coherent state is very particular, the inequalities discussed above can be further reduced. In Figure 6a–c, the allowed values for the sum of the square areas for the qubits ${\hat{\rho }}^{\left(A\right)}$, ${\hat{\rho }}^{\left(B\right)}$, and ${\hat{\rho }}^{\left(D\right)}$, defined by the coherent state |ζ〉, are plotted in terms of the mean values $\langle {\hat{J}}_{y}\rangle$ and $\langle {\hat{J}}_{z}\rangle$. As can be seen, the possible values for these areas satisfy the condition in Equation (10). In Figure 6d, the sum *S* of the areas is also evaluated and the limits (9/2,8.095) can be checked.

Finally, one can conclude that the conditions in Equations (35) and (36) can be used as a control to check the experimental measurement of the mean values of the observables ${\hat{J}}_{x}$, ${\hat{J}}_{y}$, and ${\hat{J}}_{z}$.

## 5. Conclusions

In this paper, we have used classical probabilities to describe quantum states, an approach which may provide a better understanding of quantum entanglement: the fact that this purely quantum phenomenon may be described by classical measurable probabilities seems remarkable. That the separability or entanglement of two-qubit systems can be described in purely classical terms has also been shown recently [24], where the classical Fisher metric on phase space is shown to give the same (qualitative) results as the quantum Fisher metric.

The definition of the Malevich squares and their areas is presented as a new approach to describe geometrically the qudit quantum state. In particular, the different limits for the sum of the square areas are obtained for general qubit and qutrit systems. We show some of the inequalities associated with the different areas for a spin-1 coherent state as an example of the applicability of our approach. The possible use of these expressions as a control for experimental data is also addressed.

By means of this probabilistic construction of quantum mechanics, we present the study of the linear entropy of general qubit and qutrit systems. In both cases, the entropy is written in terms of classical probability distributions, and their geometrical interpretation is discussed. In the qutrit case, one can see that the linear entropy of the system is determined by the sum of the entropies of its component qubits.

In addition, we constructed in explicit form the density matrix of some separable and entangled states of two qubits in terms of fair classical probability distributions. We obtained the characteristics of the entanglement, such as the concurrence and numeric logarithmic negativity, as functions of the probability distributions. The paradigmatic examples of the entangled states correspond to eigenstates of degenerate two-qubit Hamiltonians, which are defined in terms of three probabilities for the qubit state or eight probabilities for the qutrit state. In the latter case, these are selected from twelve dichotomous probability distributions. In a future work, we extend the procedure given here to multipartite systems.

We presented the geometrical picture of the entanglement in terms of triads of squares and found the areas of the squares for entangled states. It is worth noting that, when there is one or two inaccessible states for the two-qubit system, its entanglement properties are determined in terms of one or two spin-1/2 probability distributions. We always found an interval for *S* that provides the possibility for an experimental checkup of the system entanglement in terms of probabilities.

To conclude, we emphasize that, in the probability representation of quantum states, completely quantum phenomena such as Bell correlations in two-qubit systems can be described using only properties of classical probability distributions associated with probability interferences [32,33,34,35] and nonlinear superposition rules [19,36,37] for the probabilities determining the qudit states.

## Appendix A. Upper Bound for the Sum of the Square Areas

Due to the requirement for qutrit to be the pure state, its density matrix reads

$$\hat{\rho }=\left(\begin{array}{ccc} 1-p_{\beta }^{2}-p_{\gamma }^{2} & \sqrt{1-p_{\beta }^{2}-p_{\gamma }^{2}}\;p_{\beta }\;e^{-i\beta } & \sqrt{1-p_{\beta }^{2}-p_{\gamma }^{2}}\;p_{\gamma }\;e^{-i\gamma }\\ \sqrt{1-p_{\beta }^{2}-p_{\gamma }^{2}}\;p_{\beta }\;e^{i\beta } & p_{\beta }^{2} & p_{\beta }\;p_{\gamma }\;e^{i(\beta -\gamma )}\\ \sqrt{1-p_{\beta }^{2}-p_{\gamma }^{2}}\;p_{\gamma }\;e^{i\gamma } & p_{\beta }\;p_{\gamma }\;e^{-i(\beta -\gamma )} & p_{\gamma }^{2} \end{array}\right)\;,$$

for a state in the spin s=1 representation,

$$|\psi \rangle =\sqrt{1-p_{\beta }^{2}-p_{\gamma }^{2}}|1\rangle +p_{\beta }e^{i\beta }|0\rangle +p_{\gamma }e^{i\gamma }|-1\rangle \;.$$

Maximizing the sum of the square areas *S* with respect to $p_{\beta }$, $p_{\gamma }$, β, and γ, we obtain the upper bound given in Equation (21) for the states determined by the parameters given by $p_{\beta }\approx 0.1685$, $p_{\gamma }\approx 0.8759$, β≈0.2749, and γ≈3.9892.
